# Analysis of the nursing effects of integrated medical and nursing care intervention in correction surgery for children with concealed penis

**DOI:** 10.1186/s12912-024-01851-x

**Published:** 2024-06-26

**Authors:** Junting Li, Qifei Deng, Shengfang Zhao, Jingjing Sun

**Affiliations:** Pediatric Urology Surgery, Anhui Provincial Children’s Hospital, Children’s Hospital of Fudan University-Anhui Campus, No. 39, Wangjiang East Road, 230051 Hefei, Anhui Province China

**Keywords:** Integrated care, Pediatric, Concealed penis, Surgical procedures, Operative, Treatment outcome

## Abstract

**Objective:**

This study aimed to analyze and explore the nursing effects of integrated medical and nursing care intervention in correction surgery for children with concealed penis.

**Methods:**

A total of 76 eligible patients with concealed penis were randomly divided into an observation group and a control group. The control group received conventional nursing care, while the observation group received integrated medical and nursing care intervention. Outcomes include pain levels, comfort status, incidence of complications, and nursing satisfaction were collected and analyzed to investigate the nursing effects of the integrated medical and nursing care model.

**Results:**

After 2/3 days of nursing intervention, the patients in the observation group had significantly lower pain scores (measured by FPS-R) compared to the control group (*P* < 0.05). The patients in the observation group also had significantly higher comfort scores (measured by Kolcabal) compared to the control group (*P* < 0.05). The incidence of complications in the observation group was significantly lower than that in the control group (2.63 vs. 23.68, *P* < 0.05). Parental satisfaction in the observation group was significantly higher than that in the control group (*P* < 0.05).

**Conclusion:**

The integrated medical and nursing care intervention in correction surgery for children with concealed penis demonstrated positive nursing effects. It effectively reduced pain, improved comfort, lowered the risk of complications, and increased parental satisfaction. This approach maximizes the role of nursing care and is recommended for clinical implementation.

## Introduction

Concealed penis is a malformation of the external genitalia that can occur due to congenital or acquired factors, resulting in the penis being buried beneath the surrounding skin tissues. This condition is primarily characterized by an excess of subcutaneous fat and poor adherence between the penile skin and the corpora cavernosa, often accompanied by abnormal connections between the penile body and the fibrous bands of the lower abdominal wall [1, 2]. The reported incidence of congenital concealed penis in China is 0.67%, making it relatively common among congenital penile disorders. While simple concealed penis has minimal impact on fertility, it can hinder normal penile development due to the constriction of subcutaneous fibrous bands, and it can occur at any stage from infancy to adolescence. The appearance of a hidden penis, being shorter and smaller, can have psychological effects on a child’s sexual development, potentially leading to the development of feelings of inadequacy. Furthermore, obese boys may suffer from concealed penis, micropenis, unstable bladder, poor body image, decrease quality of life, anxiety and sexual problems [3, 4]. Moreover, if left untreated, it can lead to inflammation of penile hair follicles, difficulty in controlling the penis during urination, and challenges in retracting the foreskin for hygiene purposes, all of which can hinder penile development. Therefore, many experts recommend prompt surgical intervention after diagnosis to prevent physical and psychological issues [5].

While surgical correction is recommended for all cases of buried penis, it is essential to consider the potential limitations of previous studies. Previous research has primarily focused on the surgical procedures and outcomes of correction surgery, with limited emphasis on nursing interventions and their effects on patient outcomes. Additionally, the psychological impact of concealed penis on a child’s sexual development and the potential role of integrated medical and nursing care in addressing these concerns have not been extensively explored.

It is crucial to address the limitations of previous studies and identify areas where further research is needed. This study aims to fill this gap by analyzing and exploring the nursing effects of integrated medical and nursing care intervention in correction surgery for children with concealed penis [6]. By focusing on the nursing aspects of patient care, including pain management, comfort status, and postoperative complications, we can gain a comprehensive understanding of the potential benefits of integrated care in improving patient outcomes.

Currently, there is no standardized nursing intervention model, and traditional nursing models are insufficient to meet the clinical nursing needs of complex psychological and physiological treatments. Integrated medical and nursing care is an advanced nursing concept that emphasizes the integration of clinical and nursing operations, considering patients’ needs holistically to improve nursing quality, facilitate rapid patient recovery, and bring treatment and care closer to the expected level of patients [7, 8]. It has gradually been applied in China in recent years and represents a new nursing model in the healthcare field [9].

The novelty of this study lies in its emphasis on the nursing effects of integrated care, which has not been extensively investigated in previous research. By examining the limitations of previous studies and highlighting the need for a comprehensive approach to patient care, we aim to contribute to the existing body of knowledge and provide valuable insights for clinical practice.

Overall, this study seeks to address the gaps in the current literature and shed light on the potential benefits of integrated medical and nursing care intervention in correction surgery for children with concealed penis. By emphasizing the limitations of previous studies and identifying the unique contributions of this research, we aim to advance the understanding of optimal care strategies for this patient population.

## Materials and methods

### Patients profile

This retrospective studywas conducted with the approval of the Institutional Ethics Committee of our hospital. The study participants consisted of children who were admitted to our department and underwent concealed penis corrective surgery between January 2019 and July 2019. The initial enrolled subject was the patient who underwent concealed penis corrective surgery during this period. After excluding subjects who did not meet the complete inclusion criteria, a total of 76 eligible patients were registered and enrolled. The patients were assigned into a study group and a control group, with 38 patients in each group based on different nursing interventions implemented. The control group received routine nursing care as per usual practice, while the study group received integrated medical and nursing care intervention.

### Inclusion and exclusion criteria

Inclusion Criteria: (1) Patients with symptoms of short penile appearance, foreskin adhesion, or only foreskin exposure, clinically diagnosed as concealed penis. (2) Patients who underwent concealed penis corrective surgery at our hospital. (3) Age of patients ranging from 5 to 9 years. (4) Availability of complete clinical data.

Exclusion Criteria: (1) Presence of genetic metabolic disorders. (2) Concurrent diagnosis of balanitis or phimosis. (3) Coexisting liver, kidney, or other organ dysfunction. (4) Presence of cognitive or communication impairments that hinder active participation in treatment and care.

### Methods

#### Conventional nursing care

Conventional nursing care includes comprehensive measures aimed at ensuring the well-being of pediatric patients undergoing concealed penis corrective surgery. These measures encompass preoperative health education for the child’s parents to enhance their understanding of the condition and alleviate any tension and anxiety. Additionally, postoperatively, timely interventions are carried out to address wound care, monitor and record vital signs, guide the child in maintaining bed rest while assisting them in adopting appropriate positions to minimize the occurrence of penile skin edema. Furthermore, the nursing care involves explaining preventive measures to both the child and their family and developing a rational dietary structure to support the child’s recovery. It also includes close monitoring of localized edema and pain in the child postoperatively. In the event of any abnormalities, immediate notification of the attending physician is essential to initiate appropriate treatment and provide necessary comfort and support.

#### Integrated medical and nursing care

Integrated medical and nursing care involves the following strategies: Formation of a multidisciplinary team with uniform training for team members to define their respective roles and make use of their clinical advantages in providing high-quality nursing services to patients. Development of comprehensive protocols tailored to the integrated medical and nursing care intervention, addressing patients’ treatment and care needs throughout their hospitalization. Strengthened communication and collaboration between medical and nursing staff. Duty nurses collaborate with attending physicians during ward rounds, and within 24 h of admission, they assess and evaluate the patient’s condition, formulate a nursing care plan, which is reviewed and signed by the head nurse, and ensure effective treatment and care. Health education planning based on various clinical pathways in the department, taking into account the specific needs of the child and family. This plan is implemented collaboratively with the child’s family and may involve distributing educational materials, multimedia presentations, and one-on-one bedside education. The goal is to increase the child’s and family’s awareness of the disease and surgical procedure, eliminate negative emotions, and build trust between medical and nursing staff and the patient and family. Psychological intervention to address negative emotions such as low self-esteem and depression that can arise due to physiological barriers caused by the disease, surgical trauma, and postoperative functional limitations. Observation and communication are used to understand the negative psychological characteristics of the child and parents and their underlying causes. The focus is on addressing the concerns and worries of the child’s parents and building a comprehensive understanding of the disease in an easily understandable way. Pain management involves closely monitoring the child’s pain response. Distraction techniques, such as playing children’s songs or cartoons, are employed to divert the child’s attention and reduce discomfort. For children with severe pain, analgesic medications are administered as per medical orders. Additionally, measures to protect the surgical site, reduce friction, ensure adequate sleep, and promote rapid physical recovery are implemented. Prevention of complications includes guiding the child to assume appropriate positions to promote relaxation of the wound tension and improve compliance. This, in turn, enhances wound microcirculation, creating favorable physiological conditions for wound healing. Drainage tube care involves properly securing urinary catheter drainage bags at the child’s pubic region, educating the child and family on the importance of indwelling catheters, and ensuring that the catheter remains in place for the necessary duration. Nursing staff also closely monitor urine color and quantity and take prompt action in case of any issues. Perineal care involves keeping the perineal area exposed and dry, wiping the anus and surrounding skin with physiological saline after bowel movements, and changing dressings as needed. After removal of the stent tube and drainage tube, the child’s glans is observed for any signs of discoloration, swelling, or tissue necrosis.

Daily nursing care intervention includes dietary guidance, with a focus on a liquid diet during the first seven days after surgery, avoiding constipation to prevent damage to the wound. Adequate daily fluid intake, balanced nutrition, regular meals, and a diet rich in easily digestible foods, such as vitamins and proteins, are emphasized. Assistance with regular body cleansing, repositioning, and maintaining dry, clean bed linens is provided.

### Observational parameters

#### Pain assessment

The Facial Pain Scale-Revised (FPS-R) [10] was used to assess postoperative pain in pediatric patients on days 1–3 after surgery. The scale has a maximum score of 10, with higher scores indicating a greater degree of pain.

#### Comfort status

The simplified Kolcaba Comfort Scale [11] was employed to investigate the overall comfort levels of all patients before and after nursing interventions. This scale comprises four dimensions: psychological (32 points), physiological (20 points), environmental (28 points), and social (32 points). Scores in each dimension are directly proportional to the patient’s comfort status.

#### Complications

Detailed records of postoperative complications were maintained for all patients, including wound infections, flap necrosis, penile swelling, local hemorrhage, and others. The total incidence rates of complications were calculated for each group and compared.

#### Parental satisfaction

The Hospital Nursing Satisfaction Scale [12] was used to assess parental satisfaction. The questionnaire was distributed and completed under the guidance of professionals. The scale consists of 23 items and is categorized as “completely satisfied,” “satisfied,” “neutral,” and “dissatisfied.” Satisfaction was calculated as follows: Satisfaction Rate = (Completely Satisfied + Satisfied + Neutral) / Total Number × 100%.

### Data analysis

Data analysis was conducted using SPSS 20.0 software. Continuous data were presented as mean ± standard deviation (${\bar x}$± s) and analyzed using the independent sample t-test. Categorical data were presented as frequencies (percentages, %) and analyzed using the chi-squared test (χ²). GraphPad Prism 8 software was used for graphical data processing. Statistical significance was defined as *P* < 0.05.

## Results

### Clinical characteristics

There were no statistically significant differences in the clinical characteristics between the two groups of pediatric patients, indicating comparability (*P* > 0.05). Please refer to Table 1 for details.

**Table 1:** Comparison of Clinical Characteristics between the Two Groups of Pediatric Patients (${\bar x}$± s)

		Observation group	Control group	t	*P*
n	-	38	38	-	-
Age (years)	Minimum	5	5	-	-
-	Maximum	9	9	-	-
-	Average	7.37 ± 1.20	7.71 ± 1.39	1.395	0.171
Weight (kg)	Minimum	18	20	-	-
-	Maximum	42	41	-	-
-	Average	30.04 ± 3.35	29.28 ± 3.17	1.01	0.313
Length of hospital stay (days)	Minimum	3	3	-	-
-	Maximum	6	6	-	-
-	Average	3.87 ± 0.78	3.84 ± 0.64	0.206	0.838
Parental education	High school	10	8	-	-
-	College	15	16	-	-
-	Bachelor’s degree	13	14	-	-

### Pain assessment

**Fig. 1:**
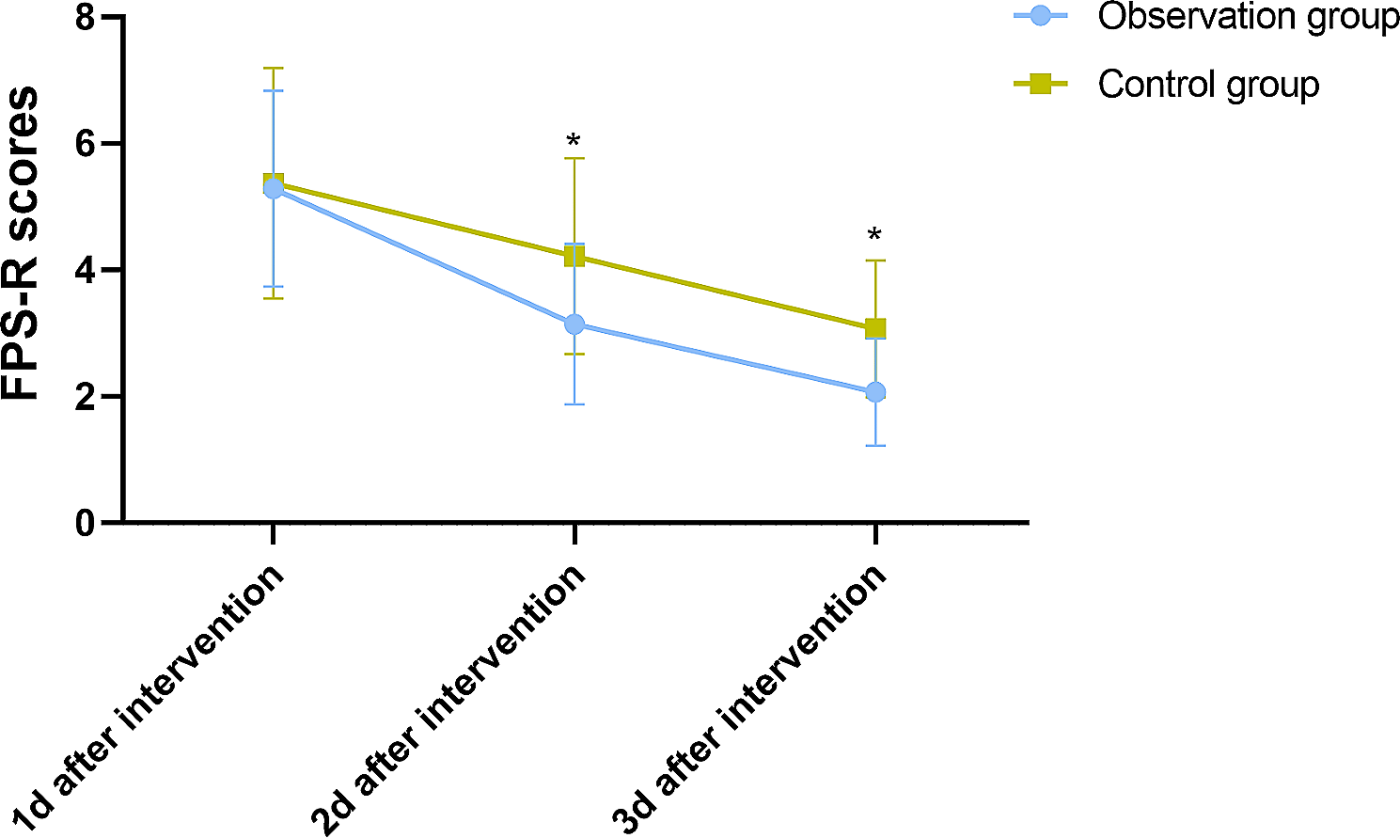
FPS-R Scores at Different Time Points for the Two Groups of Pediatric Patients. Note: * Indicates a statistically significant difference between the two groups (*P* < 0.05)

The pain assessment results demonstrated significant improvements in the observation group following the implementation of nursing intervention. The average pain scores decreased from 5.28 ± 1.55 on the first day to 3.14 ± 1.27 on the second day and further decreased to 2.07 ± 0.85 on the third day. In contrast, the control group showed less pronounced reductions in pain scores, with scores of 5.37 ± 1.82, 4.22 ± 1.55, and 3.07 ± 1.08 on the respective days (Fig. 1). Importantly, the observation group had significantly lower pain scores than the control group on the second and third days after the nursing intervention, indicating the effectiveness of the interventions in managing pain. These findings have clinical significance as they highlight the positive impact of nursing interventions in alleviating pain and improving the overall well-being of pediatric patients.

### Comfort status

**Fig. 2:**
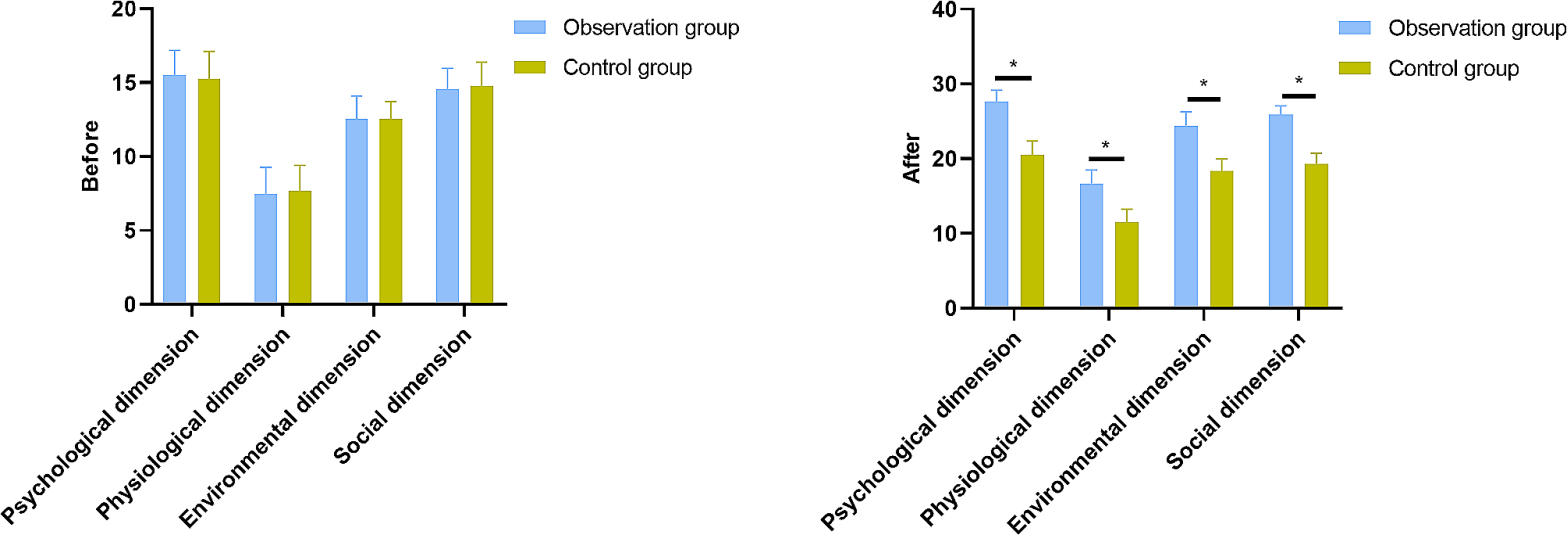
Kolcaba Scores before and after Nursing Intervention for the Two Groups of Pediatric Patients. Note: * Indicates a statistically significant difference between the two groups, *P* < 0.05

The comfort status of the observation group significantly improved across various dimensions following the nursing intervention. Before the intervention, the observation group reported comfort scores in the psychological, physiological, environmental, and social dimensions as follows: psychological (15.51 ± 1.68), physiological (7.47 ± 1.81), environmental (12.54 ± 1.55), and social (14.56 ± 1.41). After the intervention, there were marked improvements in comfort scores in all dimensions: psychological (27.65 ± 1.54), physiological (16.65 ± 1.84), environmental (24.42 ± 1.84), and social (25.89 ± 1.18). In contrast, the control group showed less improvement in comfort scores after the intervention: psychological (20.56 ± 1.81), physiological (11.51 ± 1.73), environmental (18.36 ± 1.61), and social (19.28 ± 1.47) (Fig. 2). The observation group had significantly higher comfort scores compared to the control group in all dimensions after the nursing intervention. These results underscore the clinical significance of nursing interventions in improving the overall comfort and well-being of pediatric patients.

### Complications

The incidence of complications was significantly lower in the observation group of pediatric patients compared to the control group (Table 2). This indicates that the implemented nursing interventions effectively reduced the occurrence of complications. The reduction in complications is clinically significant as it demonstrates the positive impact of nursing interventions in promoting patient safety and well-being. These findings highlight the importance of comprehensive nursing care in preventing and managing complications, leading to improved clinical outcomes and better patient experiences.

**Table 2:** Comparison of Complication Incidence between the Two Groups of Pediatric Patients (%)

	Observation group	Control group	X^2 _^	*P*
n	38	38	-	-
Wound infection	0	3	-	-
Flap necrosis	1	2	-	-
Preputial swelling	0	1	-	-
Local bleeding	0	3	-	-
Total incidence rate	2.63	23.68	7.370	0.007

### Parental satisfaction

Parental satisfaction was significantly higher in the observation group of pediatric patients compared to the control group (Table 3). This suggests that the implemented nursing interventions positively influenced the satisfaction levels of parents. Higher parental satisfaction is of clinical significance as it reflects the quality of care provided and the effectiveness of nursing interventions in meeting the needs and expectations of patients and their families. The improved parental satisfaction resulting from the nursing interventions indicates a positive healthcare experience, increased confidence in the healthcare team, and better adherence to treatment plans. These findings emphasize the importance of patient-centered care and the positive impact that nursing interventions can have on the overall healthcare experience of pediatric patients and their families.

**Table 3:** Comparison of Parental Satisfaction between the Two Groups of Pediatric Patients (%)

	Observation group	Control group	X^2 _^	*P*
n	38*23	38*23	-	-
Very satisfied	318	196	-	-
Satisfied	493	505	-	-
Neutral	40	121	-	-
Dissatisfied	23	52	-	-
Satisfaction rate	92.79	80.21	59.274	0.001

## Discussion

One of the main findings of this study was the significant reduction in pain scores among patients in the observation group who received integrated medical and nursing care intervention. It is possible that the combination of pharmacological interventions, such as analgesic medications, and non-pharmacological interventions, such as distraction techniques and relaxation exercises, synergistically contributed to pain relief [12–16]. Moreover, the provision of emotional support and effective communication by the nursing staff may have positively influenced the psychological well-being of the patients, thereby reducing their perception of pain. Future studies could explore the specific mechanisms through which these nursing interventions modulate pain perception and identify the most effective combinations of interventions.

Moreover, the significantly higher comfort scores observed in the observation group also warrant further investigation into the underlying mechanisms. It is plausible that the integrated medical and nursing care intervention addressed not only the physical comfort of the patients but also their psychological and emotional well-being. The provision of individualized care plans, tailored to the specific needs and preferences of each patient, may have contributed to a sense of security and improved comfort [17, 18]. Additionally, the active involvement of the patients’ families in the care process, including education and support, could have positively influenced the overall comfort experienced by the patients. Future studies could explore the specific components of the integrated care intervention that have the greatest impact on patient comfort and further elucidate the mechanisms involved.

In addition, the significantly lower incidence of complications in the observation group suggests that the integrated medical and nursing care intervention may have contributed to improved surgical outcomes [18–21]. A comprehensive nursing care approach, including close monitoring of vital signs, early detection and management of complications, and meticulous wound care, likely played a crucial role in preventing and minimizing adverse events. The involvement of the nursing staff in the early identification of potential complications and the prompt initiation of appropriate interventions may have contributed to the reduced incidence of complications. Future studies could investigate the specific nursing interventions that are most effective in preventing complications and explore the mechanisms through which these interventions exert their protective effects.

It is worth noting that previous studies have primarily focused on the surgical aspects of correction surgery for concealed penis, with limited attention given to nursing interventions and their impact on patient outcomes [12, 21–23]. Therefore, the current study adds novelty to the existing literature by highlighting the nursing effects of integrated care in this specific patient population. By emphasizing the limitations of previous studies and focusing on the unique contributions of this research, we provide valuable insights into the comprehensive management of children with concealed penis [21, 24].

Although this study has provided important findings, there are certain limitations that should be acknowledged. Firstly, this study was conducted at a single center with a relatively small sample size, which may restrict the generalizability of the results. Future research involving multiple centers and larger sample sizes is needed to validate these findings. Additionally, the follow-up period in this study was relatively short, preventing the assessment of long-term outcomes. Long-term studies are necessary to evaluate the sustainability and durability of the observed nursing effects. Furthermore, this study focused on exploring the nursing effects of integrated care in correction surgery for concealed penis, without delving into the specific mechanisms underlying the interventions. Future research could further investigate the mechanisms through which these nursing interventions exert their effects. This could involve examining the impact of individualized care plans, emotional support, and involvement of the patients’ families on patient outcomes. Understanding the specific mechanisms involved will enhance our understanding of the optimal nursing care strategies for children undergoing correction surgery for concealed penis. Moreover, although this study demonstrated significant nursing effects, it is important to consider potential confounding factors that were not controlled for. Factors such as socioeconomic status, pre-existing medical conditions, and variations in surgical techniques among different surgeons may have influenced the outcomes. Future studies could incorporate more rigorous study designs, such as randomized controlled trials, to minimize these potential confounders and provide more robust evidence. Moreover, the pain assessment relied on self-reported pain scores using the FPS-R (Faces Pain Scale-Revised) scale. Self-reported pain scores are inherently subjective and can be influenced by individual interpretation and perception. Additionally, the comfort status evaluation was based on the Kolcaba Comfort Questionnaire, which also relies on subjective responses from the participants. Subjective measures can be influenced by various factors such as individual expectations, cultural differences, and personal biases, which may introduce some degree of variability in the results. Another limitation is that complications and parental satisfaction were assessed using subjective measures. The incidence of complications and parental satisfaction were likely based on the perceptions and opinions of the patients’ parents or caregivers, and their responses may be influenced by their own experiences, expectations, and subjective interpretations. Lastly, the study primarily focused on the short-term outcomes and did not explore the long-term psychosocial implications of concealed penis and the impact of integrated care on these aspects. Future research could investigate the long-term psychological effects, sexual development, and quality of life outcomes in children with concealed penis who receive integrated medical and nursing care, and consider incorporating objective measures alongside subjective assessments. Objective measures such as physiological indicators, clinical observations, or objective pain assessment tools could provide a more comprehensive and balanced evaluation of the outcomes. Additionally, employing independent assessors who are blinded to the intervention group could help minimize potential bias and enhance the reliability of the results [25].

In conclusion, the findings of this study support the significant nursing effects of integrated medical and nursing care intervention in correction surgery for children with concealed penis. This comprehensive approach, which includes pain management, improved comfort, and reduced complications, demonstrates the importance of nursing care in optimizing patient outcomes. The novel contributions of this research shed light on the potential benefits of integrating nursing interventions into the management of children with concealed penis and provide a foundation for further studies in this field.

## Data Availability

All data generated or analysed during this study are included in this published article.
